# Somatic copy number profiling from hepatocellular carcinoma circulating tumor cells

**DOI:** 10.1038/s41698-020-0123-0

**Published:** 2020-07-02

**Authors:** Colin M. Court, Shuang Hou, Lian Liu, Paul Winograd, Benjamin J. DiPardo, Sean X. Liu, Pin-Jung Chen, Yazhen Zhu, Matthew Smalley, Ryan Zhang, Saeed Sadeghi, Richard S. Finn, Fady M. Kaldas, Ronald W. Busuttil, Xianghong J. Zhou, Hsian-Rong Tseng, James S. Tomlinson, Thomas G. Graeber, Vatche G. Agopian

**Affiliations:** 1 Department of Surgery, University of California, Los Angeles, Los Angeles, CA USA; 2 Department of Surgery, Veteran’s Health Administration, Greater Los Angeles, Los Angeles, CA USA; 3 Department of Molecular, Cellular, and Integrative Physiology, University of California, Los Angeles, Los Angeles, CA USA; 4 PacGenomics, llc, Los Angeles, CA USA; 5 Department of Molecular and Medical Pharmacology, University of California, Los Angeles, Los Angeles, CA USA; 6 Department of Medicine, Division of Hematology/Oncology, University of California, Los Angeles, Los Angeles, CA USA; 7 California NanoSystems Institute, University of California, Los Angeles, Los Angeles, CA USA; 8 Department of Pathology and Laboratory Medicine, University of California, Los Angeles, Los Angeles, CA USA; 9 Jonsson Comprehensive Cancer Center, University of California, Los Angeles, Los Angeles, CA USA

**Keywords:** Prognostic markers, Molecular medicine

## Abstract

Somatic copy number alterations (SCNAs) are important genetic drivers of many cancers. We investigated the feasibility of obtaining SCNA profiles from circulating tumor cells (CTCs) as a molecular liquid biopsy for hepatocellular carcinoma (HCC). CTCs from ten HCC patients underwent SCNA profiling. The Cancer Genome Atlas (TCGA) SCNA data were used to develop a cancer origin classification model, which was then evaluated for classifying 44 CTCs from multiple cancer types. Sequencing of 18 CTC samples (median: 4 CTCs/sample) from 10 HCC patients using a low-resolution whole-genome sequencing strategy (median: 0.88 million reads/sample) revealed frequent SCNAs in previously reported HCC regions such as 8q amplifications and 17p deletions. SCNA profiling revealed that CTCs share a median of 80% concordance with the primary tumor. CTCs had SCNAs not seen in the primary tumor, some with prognostic implications. Using a SCNA profiling model, the tissue of origin was correctly identified for 32/44 (73%) CTCs from 12/16 (75%) patients with different cancer types.

## Introduction

Somatic copy number alterations (SCNAs) are found in 90% of solid tumors and are increasingly recognized as playing a vital role in activating oncogenes and inactivating tumor suppressors through changes in gene dosage and structure^1^. Newer sequencing methods and large-scale genetic studies indicate that SCNAs affect a larger fraction of the genome in cancer than any other somatic alteration^1^. In fact, SCNAs have recently been shown to provide the largest contribution to a pan-cancer tumor classification model, greater than that provided by transcriptome and methylome alterations^2^. Research has also found that larger SCNAs, such as whole chromosome, arm, and cytoband length events, are likely more important than focal SCNAs in the development of cancer^1,3^. These larger SCNAs are easily detectable using next-generation sequencing (NGS) techniques, and result in a very robust and reproducible signal^4^. These favorable characteristics make NGS-based SCNA profiling an ideal molecular study for limited template samples like fine needle aspirates or circulating tumor cells (CTCs).

CTCs, cells of tumor origin that circulate in the blood, are a promising new biomarker for many solid tumors^5,6^. As potential metastatic precursors^7^, CTCs are thought to represent the subclones of the primary tumor that are more invasive^8,9^. Their presence is associated with a higher risk of recurrence and mortality in many cancer types and across all stages of disease^5,6^. Recently, advances in CTC isolation and sequencing has evolved to the point that CTCs may soon serve as a form of “liquid biopsy” for cancer patients, tests done on the blood to look for cancer cells from a tumor that are circulating in the blood^8^.

The majority of studies on the molecular characterization of CTCs in solid tumors have focused on the detection of actionable somatic point mutations through targeted or exome sequencing. However, due to the combination of cost, analysis time, and risk of false positives at typical exome sequencing depths, exome sequencing of CTCs has not seen significant clinical use. In contrast, SCNA profiling of CTCs via low-resolution NGS-based whole-genome sequencing (WGS) has recently been described and has the advantage of offering a robust signal at a significantly lower cost than exome sequencing^4^. Given the recent advances in our understanding of the importance of SCNAs for cancer prognosis and treatment, SCNA analysis of CTCs has significant potential as a biomarker^10–13^.

To better understand the feasibility and potential of SCNA profiling of CTCs as a liquid biopsy, we attempted to address several important methodological questions. These included validating the tumor origin of the isolated CTCs, as well as demonstrating the reliability and reproducibility of the assays that are utilized for CTC characterization^14^. We recently developed a HCC-specific CTC isolation method and demonstrated its efficacy for isolating and enumerating CTCs in hepatocellular carcinoma (HCC)^15^. We have now developed a modification to that assay that allows for low-resolution WGS of the isolated CTCs using whole-genome amplification (WGA) and NGS. Using this methodology, our current study investigates NGS-based SCNA profiling of CTCs as a potential molecular biomarker for HCC patients.

To that end, we performed a pilot study to investigate SCNA profiling of HCC CTCs isolated immediately prior to surgical resection, and compared them to the SCNA profiles from the surgically resected HCC tumor tissue, peritumoral non-cancerous liver, and genomic DNA from pooled white blood cells. With a robust analysis of multiple DNA sources from individual patients, we aimed to extensively validate the tumor origin of the isolated CTCs, to establish that CTC-derived SCNA profiles can serve as surrogates of the underlying molecular alterations in the primary HCC tumor, and to demonstrate the utility of a CTC-derived SCNA assay as a clinically useful liquid biopsy in HCC.

## Results

### Patient characteristics and sample collection

**Fig. 1:**
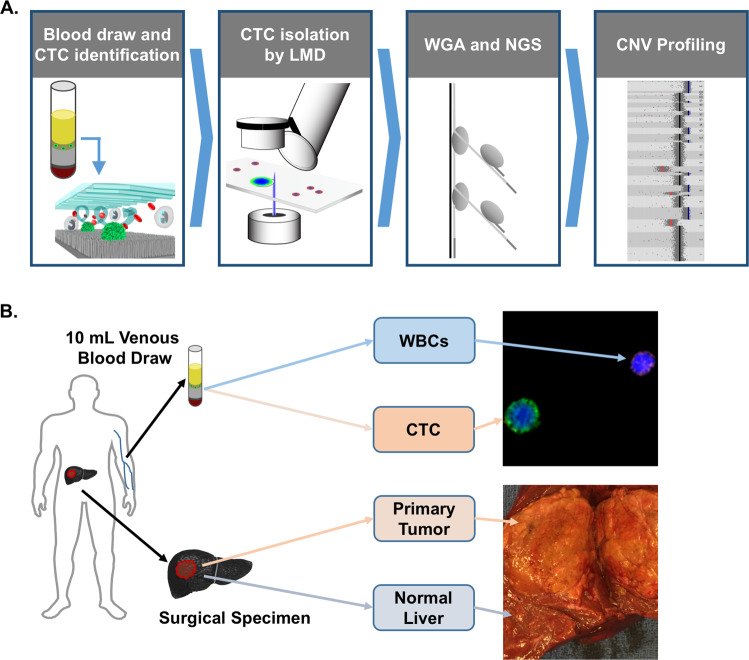
Experimental design of the study. **a** Workflow used in the study for CTC isolation and SCNA profiling using the NanoVelcro assay. Following gradient centrifugation, HCC CTCs are selectively captured on the NanoVelcro surface for identification and subsequent isolation by LMD. CTC samples undergo WGA and QC prior to low-resolution whole-genome sequencing for SCNA profiling. **b** A total of four specimens are obtained from each patient. From peripheral blood, germline genomic DNA is obtained from the bulk buffy coat layer. Circulating tumor cells are obtained and sequenced as described in (**a**). From the surgically resected specimen, a sample of both the primary tumor and the peritumoral normal liver are obtained and sequenced.

Ten consecutive patients undergoing surgical resection of HCC from our ongoing prospective HCC biomarker protocol between March, 2016 and January, 2017 were included for analysis. The clinical, laboratory, radiologic, and treatment characteristics of the ten patients are summarized in Supplementary Table 1. Nine of the patients underwent resection of a primary liver tumor and one patient underwent resection of a retroperitoneal metastasis that developed 10 years after liver transplantation for HCC (patient H167). Nine of the patients had cirrhosis at the time of blood draw, with the majority of these patient’s cirrhosis resulting from HCV infection. All patients had DNA from whole blood, peritumoral normal liver, HCC tumor tissue, and CTCs available for molecular analysis (Fig. 1b).

### CTC enumeration and sequencing

CTC enumeration revealed a median of four (IQR: 2–11, range: 1–16) CTCs per 4-mL VB. Multiple displacement amplification-based WGA was successfully performed on CTC samples, and low-resolution WGS performed on a NextSeq 500. Sequencing reads for the CTC samples were processed using a variable bin algorithm specifically designed for low-resolution single cell copy number determination^4,16^. We obtained an average of 0.88 million uniquely mapped reads (range: 0.51–1.9 million uniquely mapped reads) from the CTC samples, with an average depth of coverage of 0.022× and a minimum read count of 0.5 million reads for CTC samples. Blood, peritumoral normal liver, and HCC tumor tissue DNA were extracted as detailed in the methods before being subjected to the same sequencing and analysis pipeline as the CTC samples, resulting in a median of 0.99 million uniquely mapped reads (IQR: 0.86–1.2 million reads, range: 0.38–2.1 million reads).

### CTC versus primary tumor SCNA profiles

**Fig. 2:**
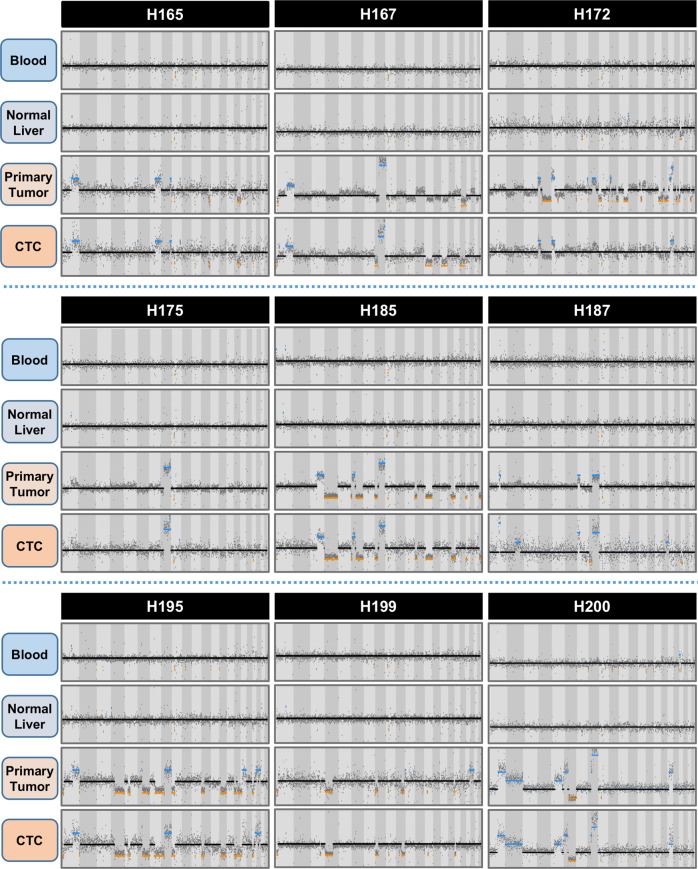
Global copy number profiles for blood, peritumoral normal liver, CTCs, and primary tumor (*n* = 8) or solitary metastasis (*n* = 1, H167) demonstrates the recapitulation of SCNAs from the tumor in CTC samples, supporting the potential of CTCs to act as a liquid biopsy of the tumor. Patient H169’s CTC (shown in Supplementary Fig. 1) STR analysis did not match that of the tumor or blood sample, suggesting contamination.

We performed STR typing of the blood, primary tumor, and CTCs to confirm that contamination had not occurred during WGA processing. While STR typing confirmed that CTCs had originated from the same individual for 9/10 patients, it revealed that the CTCs for patient H169 were likely from a different source and this patient was excluded from further analyzes (Supplementary Fig. 1). Next, whole-genome SCNA profiles were obtained by visualizing the copy number state at each 250-kbps bin along the entire genome for the DNA from whole blood, normal liver, the primary tumor and CTC samples (Fig. 2). For the nine patients with confirmatory STR typing, the whole-genome SCNA profiles were compared with determine if CTCs exhibit the somatic SCNAs found in the primary tumor. Inspection of the copy number profiles clearly demonstrates the recapitulation of the somatic changes of the primary tumor by the CTCs, helping to establish the tumor origin of the CTCs. Most of the SCNAs seen in Fig. 2 are in regions of the genome previously identified as somatic SCNAs associated with HCC (Supplementary Table 2). Overall, the median sensitivity of CTC SCNA profiling for identifying individual gene-level SCNAs in the primary tumor for all patients was 91% (IQR: 0.87–0.98), while the specificity was 97% (IQR: 0.94–0.99). We confirmed the SCNA profiles found by NGS using array CGH for a subset of six patients with sufficient DNA available, and again found that the CTCs recapitulated the somatic changes found in the primary tumor (Supplementary Fig. 2).

### CTC clustering analysis

**Fig. 3:**
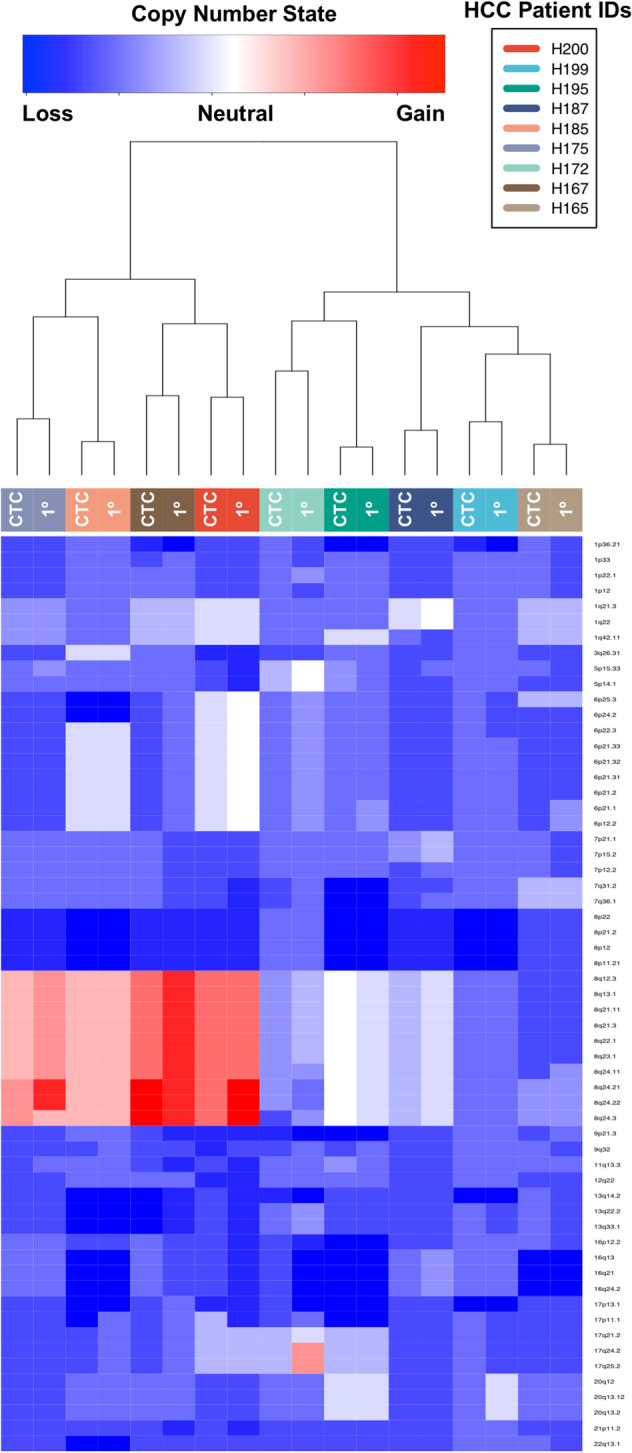
Heatmap showing copy number profiles at 59 loci frequently amplified or lost in HCC. Gain (red) or loss (blue) at each of the 59 cytobands (identified on the right of the heatmap) in both CTC and tumor samples from all nine patients that passed STR analysis are shown. Unsupervised hierarchical clustering demonstrates clustering of CTC–tumor pairs for all nine patients. CTC—circulating tumor cell, 1°—Primary tumor.

To statistically verify the visualized results of Fig. 2, we developed a 59 loci panel of previously identified regions or genes amplified or deleted in HCC (Supplementary Table 2)^17–24^. We compared the segmented, normalized copy number data of CTCs and primary tumors at the 59 loci in the panel, and found frequent somatic changes for all patients, consistent with previous studies of HCC tumors (Fig. 3). Unsupervised hierarchical clustering of the CTC and tumor copy number values demonstrated that CTC samples clustered with their respective primary tumor for all nine patients. Analysis of the global copy number profiles by a Spearman correlation matrix (Supplementary Fig. 3**)** demonstrated that the CTC SCNA profiles shared an average of 95% (IQR: 86–97%) of the gains and losses found in the primary tumor.

### CTC SCNA profile reproducibility

To assess the reproducibility of our assay, we isolated and sequenced seven independent CTC samples of 3–4 cells from a single patient (patient H195). Principle component analysis of the whole blood, normal liver, primary tumor, and all seven CTC samples for this patient revealed that five of the CTC SCNA profiles clustered closely with the primary tumor (Supplementary Fig. 4A). One CTC sample (CTC 7) clustered with the peritumoral normal and blood samples and on examination of CTC 7’s copy number profile, it demonstrates a substantially different profile than that of the other CTCs, and is closer to that of the germline DNA, likely representing either a false positive CTC call or contamination with normal human DNA (Supplementary Fig. 4B). One additional CTC sample (CTC.4) clustered on its own, and examination of its copy number profile revealed significant discordance with all other samples (Supplementary Fig. 4B).

### CTC SCNA sequencing as a molecular biomarker

**Fig. 4:**
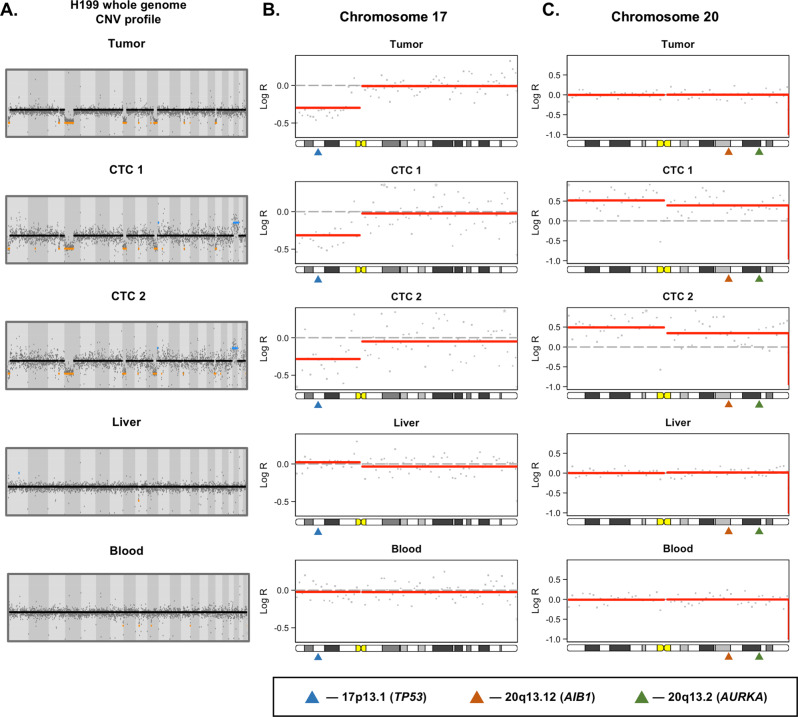
Comparison of low-resolution whole-genome copy number profiles for primary tumor, CTCs 1 and 2, normal liver, and whole blood reveals reproduction of the majority of the SCNAs from the primary tumor in both CTC samples. **a** Whole genome SCNA profiles for the primary tumor as well as the two CTC samples. **b** Chromosome 17p loss is seen in the primary tumor and both CTC samples, but not the normal liver or whole blood. The location of the tumor suppressor TP53 gene, the most frequently mutated or lost gene in HCC, is indicated by the blue triangle. **c** Chromosome 20 amplification was seen in both CTC samples but not in the primary tumor. Chromosome 20 amplifications are a recurrent somatic copy number alteration in HCC associated with 2 important oncogenes. Overexpression of *AIB1* (orange triangle) is frequently described in HCC, and has previously been associated with invasiveness. In addition, recent research has demonstrated that the oncogenic effects of *MYC* dysregulation, a common occurrence in HCC, require overexpression of *AURKA* (green triangle) for stabilization. Furthermore, in p53-altered HCC patients, the MYC-AURKA complex is an actionable drug target based on preclinical studies.

To investigate the potential clinical relevance of our CTC SCNA profiling to act as a surrogate for the primary tumor of interest, we explored our ability to detect prognostic or actionable copy number changes identified by prior studies of HCC genetics. One such example is illustrated in Fig. 4. Patient H199 is a 64-year-old male with cryptogenic cirrhosis and a 9.0 cm segment 2–3 AFP-nonproducing lesion who underwent a left hepatectomy. On pathologic examination, his tumor was found to be moderately differentiated with evidence of microvascular invasion but no macrovascular invasion.

Examination of the copy number profile from the primary tumor revealed copy number loss at chromosome 1p, 4p, 8p, 10q and 17p (Fig. 4a). Of particular note is the chromosome 17p loss, as it contains the well-known tumor suppressor *TP53* gene, the most frequently mutated or lost gene in HCC (Fig. 4b)^17^. When examining the two CTC samples from this patient, all of the losses found in the primary tumor were detected; however, an additional amplification of chromosome 20 was detected from both CTC samples (Fig. 4c). Chromosome 20 amplifications are a recurrent somatic SCNA in HCC associated with 2 important oncogenes^25–29^. Overexpression of *AIB1* is frequently described in HCC, and has previously been associated with invasiveness and sensitivity to the sorafenib therapy^25,29^. In addition, recent research has demonstrated that the oncogenic effects of *MYC* dysregulation, a common occurrence in HCC, require overexpression of *AURKA* for stabilization^28^. This discovery led to recent preclinical study which found that in p53-altered HCC patients, the *MYC*-*AURKA* complex is an actionable drug target^26^. While an intriguing finding, these studies are all preclinical at this time. We further investigated if any recurrent SCNAs were found at a higher frequency in the CTCs when compared with the primary tumor samples. No arm or chromosome level SCNAs were identified; however, we did find losses of cytoband 19p12, 2q33.2, 4p14, and 5q13.2 as well as amplifications of 11p15.5 more frequently in the CTCs than in the primary tumor samples.

### Cancer type classification using SCNA profiles

**Fig. 5:**
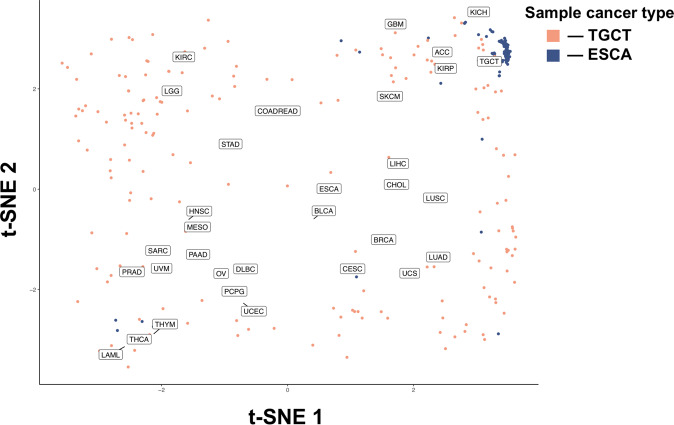
Copy number alterations at all genes for the 32 cancer types were transformed using t-SNE into a 2-dimensional space. The mean of all samples for each cancer type is plotted as well as the individual samples for the best clustering (TGCT—Blue) and worst clustering (ESCA—Pink) cancer types. All individual samples are plotted by cancer type in Supplementary Fig. 5.

As CTCs are universally shed by all tumor types, and CTCs demonstrate similar SCNA patterns to the primary tumor, we investigated the ability of CTC SCNAs to determine the site of origin of the primary tumor. To do so we obtained whole-genome copy number data for 10,478 samples from 31 tumor types available in the TCGA dataset^30^. The copy number state at 268 cancer-associated cytobands for all samples of the 31 cancer types was evaluated visually through 2-dimensional transformation using t-SNE (Fig. 5; Supplementary Fig. 5)^31^. While some cancers such as glioblastoma multiforme (GBM) or testicular germ cell tumors (TGCT) demonstrated clear clustering, others such as bladder cancer or esophageal cancer (ESCA) had samples scattered across the t-SNE space with no clear clustering identified. We used the copy number state of each sample to calculate three whole-genome metrics to help with tumor origin classification: a chromosomal instability number (CIN) score as well as the two t-SNE dimension variables. We then trained a random forest model to predict the tumor site of origin on the training set (80% of samples) and obtained an overall model accuracy of 0.58 (95% CI: 0.56–0.60) for the test set. Analysis of the misclassification rate revealed that many misclassifications were occurring between expected classes such as low-grade glioma and GBM or between different types of kidney tumors (KICH, KIRC, and KIRP). In addition, several cancer types did not demonstrate a significant site-specific copy number pattern using either t-SNE or random forest classification. Thus, to improve our model we eliminated poorly clustering tumor types (*n* = 10, Supplementary Fig. 5), and grouped the remaining 21 cancer types into 11 cancer classes based on data from prior studies on cancer subtype classification (Supplementary Table 3)^2^. Repeating the modeling on the 11 cancer class model resulted in an overall accuracy of 0.83 (95% CI: 0.78–0.88) with individual cancer class balanced accuracies ranging from 0.75–0.98.

### CTC cancer type classification

**Fig. 6:**
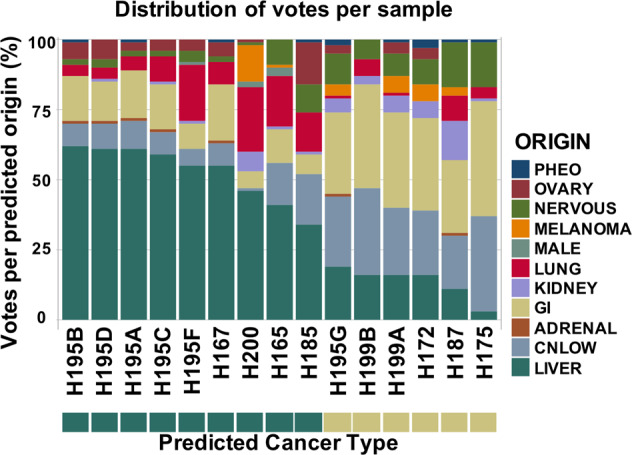
Cancer class prediction based on SCNA profile for each of the 15 HCC CTC samples using the classification model. 9/15 (60%) of CTCs were correctly classified as being HCC, while 6/15 (40%) were classified as being from a GI source. 5/9 (56%) patients had at least one CTC classified as being from a HCC tumor.

Given the circulating nature of CTCs we next sought to determine if CTC SCNA profiles could be used to determine the tissue of origin from which the CTCs originated. A total of 9/15 (66%) HCC CTCs from our patients were correctly classified, and 5/9 (56%) patients had at least 1 CTC sample identify HCC as the tissue of origin (Fig. 6). To further investigate our model, we searched for additional CTC SCNA studies but identified only a single study for which data were available. This study by Ni et al. looked at 29 CTC samples from 7 lung cancer patients^10^. For these lung cancer CTCs, the model identified the tissue of origin correctly for 23/29 (79%) CTCs and all 7 patients had at least 1 CTC sample identifying lung adenocarcinoma as the cancer type correctly (Supplementary Fig. 6). Overall, our model correctly identified the cancer type for 32/44 (75%) CTCs for the two cancer types.

## Discussion

Blood-based “liquid” biopsies hold numerous potential benefits over traditional percutaneous or surgical biopsies such as reduced risk, cost, and patient discomfort. Furthermore, they are increasingly recognized as a necessary component of a precision oncology treatment strategy, given the ongoing need for tumor tissue as the tumor adapts to new therapies^32^. Despite these benefits, liquid biopsies have yet to enter clinical practice due to issues including reproducibility and applicability for molecular analysis^33^. One reason may be that most molecular liquid biopsy studies to date have investigated detection of point mutations, due to the much larger number of actionable mutations compared with actionable SCNAs. However, accurate mutation calling from single cells or limited template samples is difficult due to the relatively high error rate involved^34,35^. In contrast, whole-genome NGS SCNA profiling of liquid biopsies results in a robust signal that is highly reproducible from as few as a couple hundred thousand reads^4,36^. Recent studies have found that SCNAs represent the largest portion of the somatic genetic changes across all cancer types^11^, may be more important driver events than somatic mutations for many cancer types^37^, and their number within a given tumor directly correlate with outcomes such as recurrence and mortality^38^. However, the specific gene-level drivers within large SCNA requires further investigation before their biological and clinical significance is established. While some cancer types have known SCNAs with therapeutic implications, such as gastric cancer and FGFR2 or breast cancer and HER2, the majority do not currently^39^. Hopefully that will change soon given the numerous ongoing studies into the clinical importance of SCNAs in many different cancer types. To that end, we sought to investigate the validity of NGS-based SCNA profiling of CTCs and to demonstrate the potential utility of such an assay for different clinical applications. The current work represents the first reported work of SCNA analysis of HCC CTCs, confirming their tumor origin and demonstrating potential prognostic importance.

Previous research into low-resolution copy number profiling has demonstrated that as few as 250–350k reads are sufficient for calling copy number events larger than 500 kbp^4^. Thus, our sequencing method, which resulted in less than a million reads per sample, potentially limits our detection of smaller focal SCNAs. However, current research indicates that whole chromosome, arm, and cytoband length SCNAs are more important in the development of cancer than smaller SCNAs, making our depth sufficient^1,3^. When run in bulk, our assay is also surprisingly affordable; a prior study using a similar protocol showed that the total reagent and sequencing costs for such a SCNA profiling assay would be as low as US$30, a significant improvement over older array CGH-based SCNA assays^4^.

A fundamental concern for molecular profiling of CTCs is ensuring that the CTCs are in fact of tumor origin and not just circulating epithelial cells^40^. We employ a strict CTC definition which helps eliminate false positive CTC calls; however, it also results in fewer CTCs captured versus other CTC platforms. Due to the relatively few cells in the resulting sample, single-cell sequencing techniques are required for analysis, which introduces errors common to those techniques^41^. Of the 16 CTC samples sequenced, only 3 (19%) of them demonstrated problems. One sample did not pass STR typing analysis indicating potential contamination, one sample demonstrated decreased signal and missing SCNAs indicative of contamination with germline DNA, and one sample had an uninterpretable SCNA profile indicative of failure at some step of the protocol. These failures are all common problems when working with single-cell sequencing and the failure rate could likely be greatly reduced by implementing clean room controls and automation^41^. For the samples that were successfully sequenced, we found that CTC SCNA profiles consistently demonstrated that the copy number changes seen in the primary tumor are also found in the CTCs. This finding makes us confident that the CTCs we sequenced likely originated from the primary tumor, and that CTCs can act as molecular surrogates of the primary tumor for SCNA profiling. We further tested the reproducibility of the assay by sequencing multiple CTC samples from a single patient and found similar CTC profiles from almost all of the CTC samples.

In addition to confirming the tumor origin of our samples, we demonstrated two further potential applications of our CTC SCNA profiling assay: identification of prognostic or targetable SCNAs and determining the tumor site of origin for CTCs. Many prognostic and targetable SCNAs were found in our CTC samples. For example, patient H199’s two CTC samples both demonstrated the chromosome 17p loss seen in the primary tumor. However, they both also showed chromosome 20 amplifications, a prognostic and potentially actionable finding^26,29^. Prior studies have demonstrated that metastases tend to arise from a single subclone of the primary tumor^42^, and that CTCs have been shown to be oligoclonal precursors of metastases in breast cancer^7^. Thus, it is plausible that CTCs originate from the more aggressive subclones of the primary tumor. While further studies are necessary to investigate this hypothesis, if true, it would lend credence to the idea that CTC-based liquid biopsies selectively sample aggressive subclones at increased risk of metastasizing. This would potentially allow CTCs to overcome the abundant tumor heterogeneity that can limit the clinical utility of traditional percutaneous biopsies for some cancer types such as HCC^43^.

Prior studies investigating the classification of tumors by site of origin have found that SCNAs are the most important genetic determinant in classification models, contributing more information than mRNA expression, miRNA expression, or DNA methylation data^2^. Our finding that some cancer types were well classified by SCNAs while others were not has previously been reported in other studies investigating SCNA-based classification^37,44^. Overall, we found that SCNA data alone could correctly identify the tumor site of origin for most cancer types, and that most of the errors were due to known similarities between some cancers such as between head and neck squamous cell and esophageal squamous cell cancers. Applying our model to CTC SCNA data we could determine the site of origin of the CTCs for the majority of samples, both from our own CTC data of HCC patients in addition to SCNA data from a prior study of lung cancer CTCs^10^. While we do see a direct clinical use for this finding, further investigation and refinement of the model could help with difficult scenarios such as identifying the site of origin for patients with tumors of unknown origin, or in cases of recurrence with multiple prior primary tumors.

We investigated the feasibility of low-resolution NGS SCNA profiling of HCC CTCs as a molecular liquid biopsy and present the potential applications of such an assay. Analysis of CTCs and primary tumor tissue demonstrated concordant alterations that were not present in the peritumoral normal liver or blood genomic DNA. This supports the potential use of CTC-derived SCNA profiling as a clinically relevant surrogate of the primary tumor. To our knowledge, this is the first paper looking at SCNA analysis of HCC CTCs and believe that future studies involving larger sample sizes will be needed to better address the clinical utility of the assay. The current study demonstrates proof-of-principle for CTC SCNA analysis, and provides a new potential method to utilize CTCs for precision oncology.

## Methods

### Patient recruitment, sample processing, and CTC isolation

We prospectively enrolled patients undergoing surgical resection of HCC under our Institutional Review Board (IRB) approved protocol at the University of California, Los Angeles (IRB #14-001932)^45^. All participants in the study provided written informed consent. Following discard of 5 mL of peripheral venous blood to prevent epithelial contamination, 10 mL of venous blood was collected in the operating room immediately prior to surgical resection into ACD solution A tubes (BD Pharmigen, Franklin Lakes, NJ), and stored at 4 °C until processed. All samples were processed within 24 h of collection. Following initial density gradient centrifugation, the buffy coat is incubated with a cocktail of biotinylated CTC capture antibodies against the HCC cell surface markers asialoglycoprotein receptor (Abcam, Cambridge, UK), glypican-3 (Santa Cruz Biotechnology, Santa Cruz, CA), and epithelial cell-adhesion-molecule (EpCAM; Cell Signaling, Danvers, MA). Following capture antibody incubation, cells are washed and re-suspended in PBS (Gibco, Carlsbad, CA) and processed on the NanoVelcro platform (Fig. 1a)^45^.

In addition to peripheral blood samples, all patients had a single radiographically apparent lesion, and had both a section of the primary tumor and a section of the peritumoral normal liver isolated and flash frozen for subsequent molecular analysis. All patients underwent a blood draw prior to surgery, with a portion of the venous blood sample being processed to obtain germline genomic DNA and the remainder used for CTC isolation. Thus, all patients had DNA from whole blood, peritumoral normal liver, primary HCC tumor tissue, and CTCs available for molecular analysis (Fig. 1b).

### P-NanoVelcro CTC chip processing, immunocytochemistry, and chip scanning

The assembly, operation, and staining of P-NanoVelcro CTC chips^45,46^ uses an electro-spin method to assemble the Poly(lactic-co-glycolic acid) (PLGA) nano-spun chips onto a laser microdissection slide (Leica, Wetzlar, Germany) with an overlaid custom polydimethylsiloxane microfluidic component and attached to a syringe-based microfluidic pump (KD Scientific, Holliston, MA). Chips are stained and CTCs identified via scanning fluorescent microscopy on a Nikon Eclipse 90i using immunocytochemistry (ICC) and NIS Elements 4.1 software. Chips are first scanned at 40× power followed by higher magnification manual imaging of candidate cells at 400× power for verification (Supplementary Fig. 7A). For the resulting multi-channel ICC image, CTCs are defined as round/ovoid cells, DAPI^+^/CD45^−^/CK^+^, with size ≥ 6 µm. WBCs are defined as round/ovoid cells, DAPI^+^/CD45^+^/CK^−^, with size ≤ 6 µm. Any cell displaying CD45 positivity greater than 2× background were excluded as CTCs. CTC enumerations are reported as total counts per 4-mL venous blood, and were performed by the same blinded researcher (S.H.).

### Laser micro-dissection

After CTC identification as outlined above, CTC chips were transferred to an ArcturusXT laser capture microdissection system (Thermo Fisher Scientific, Waltham, MA) attached to a Nikon Eclipse Ti microscope, and the CTCs were isolated into CapSure HS Caps (Thermo). Cell transfer to the cap was confirmed by light microscopy, and cells re-suspended in 4-µL PBS using a sterile pipette tip (Supplementary Fig. 7C). All candidate CTC cells from a single PLGA slide (equivalent to 2-mL of whole blood) were re-suspended on to a single cap with the exception of patients who had >5 CTCs per slide (*n* = 2).

### Whole-genome amplification and genomic analysis

Re-suspended cells were lysing and whole-genome DNA amplified using the REPLI-g Single Cell Kit (Qiagen) using the manufacturer’s recommended protocol. Amplified DNA was purified by AMPure XP beads (Beckman Coulter, Brea, CA) using the manufacturer’s recommended protocol resulting in 25 µL of purified WGA product.

Purified WGA products were sheared to generate DNA fragments averaging 350 bps by sonication (Covaris, Woburn, MA). Sonicated DNA was cleaned, end-repaired, ligated, and amplified using the KAPA DNA Library Preparation Kit (KAPA Biosystems, Wilmington, MA) according to the manufacturer’s protocol. Sequencing was performed on an Illumina NextSeq 500 (Illumina, San Diego, CA) using 75 bp paired end reads (2 × 75 bp).

### Short tandem repeat (STR) analysis

To eliminate contamination as a confounding factor, we performed STR analysis of all CTC samples and compared it to that of the primary tumor and whole blood with the GenePrint 10 v1.1 system (Promega, Madison, WI) using the manufacturer’s recommended protocol: a 10 ng aliquot of template DNA was added to the amplification master mix and amplified for 30 cycles on a GeneAmp PCR System 9700 thermal cycler (Thermo). Fragments were analyzed on a AB 3500 Genetic Analyzer with POP-4 Polymer (Applied Biosystems, Foster City, CA) and visualized using GeneMapper 5 software (Applied Biosystems).

### Array comparative genomic hybridization

Sample DNA (CTC, whole blood, peritumoral normal liver, and tumor tissue) and reference DNA (Agilent, Santa Clara, CA) were differentially labeled with cyanine-3 (CY3) and cyanine-5 (Cy5) dyes using the GenetiSure Amplification and Labeling Kit (Agilent) according to the manufacturer’s protocol. Purified labeled DNA samples were prepared for hybridization, which took place on Agilent 8×60 K CGH microarray slides at 67 °C for 6 h. Following the hybridization, the slides were scanned using the Agilent SureScan Microarray Scanner (Agilent). Microarray images were analyzed using the Agilent CytoGenomics software (Agilent) and the Microarray text files were analyzed using R version 3.3.2 and the packages rCGH, limma, agilp, and snapCGH.

### SCNA analysis from WGS data

The low pass WGS data was processed for SCNA analysis using Ginkgo with a variable bin size of 250 kbps and simulating bins of 76 bp reads mapped with bowtie^16^. CTC samples demonstrated amplification bias due to GC content and mappability that was corrected using loess smoothing^4,16^. To evaluate the ability of CTC SCNA profiles to identify the tumor type of origin, additional lung cancer CTC data from the study by Ni et al. was obtained and processed using the same methodology as for the CTC samples from our study^10^. The resultant matrix of binned SCNA values was similarly transformed to a SCNA per gene matrix based on gene–bin overlap using biomaRt^47^.

### TCGA copy number analysis and cancer site of origin classification

TCGA copy number profiles for all cancer types listed in Supplementary Table 4 were obtained from the Broad Institute’s Firebrowse TCGA data version 2016_01_28 using FirebrowseR^30^. A total of 10,478 samples from 32 tumor types were used (Supplementary Table 4). The high dimensional patient × gene dataframe was reduced transformation from a patient × gene dataframe to a patient × cytoband dataframe using biomaRt^47^. The resulting 556 cytobands were then reduced to just 274 cytobands that were previously associated with global and cancer type specific SCNAs based on prior pan-cancer SCNA analysis^1^.

Two pan-genomic variables were created to assist with classification. First, a chromosomal instability number (CIN) score, defined as the absolute value of all SCNAs for each sample. Next, two t-SNE variables were created by dimensional transformation using the Rtsne package implementation of the t-distributed Stochastic Neighbor Embedding (t-SNE) algorithm^31,48^. Both the CIN score and the two t-SNE values for all samples were added to the final dataset used in the classification model.

Tumor site of origin classification was performed on the TCGA data using a random forest classifier. We eliminated cancer types with poor classification accuracy and grouped similar cancer types into cancer classes (e.g., grouping HCC and choleangiocarcinoma) resulting in a final model with 21 cancer types and 11 cancer classes (Supplementary Table 3)^2^. The resulting 11 cancer classes were then used to train a random forest model (*n* = 500 trees) based on the TCGA SCNA data. Parameter tuning was performed by a repeated cross-validation approach and the final model verified for overall accuracy using the test set. This final model was then used to classify the CTC samples from both this study as well as the previously published lung cancer CTC study^10^.

### Statistical analysis

Statistical analysis and visualization were performed in R (version 3.3.2). Categorical variables were summarized as frequencies and percentages while continuous variables were summarized as medians and interquartile ranges (IQR). All CTC numbers are reported as whole numbers in 4-mL of venous blood.

The “gplots” package was used for unsupervised hierarchical clustering of SCNA profiles from CTC and primary tumor samples using complete linkage and Euclidean distance metric^49^. The correlation of the SCNA pattern of the CTCs to that of the matched primary tumor samples was calculated using the Pearson’s correlation.

## Supplementary information

Supplemental Methods, Tables, and Figures

## Data Availability

CTC next-generation sequencing data are publicly available in the NCBI Sequence Read Archive (SRA) under the accession number PRJNA630090. The other datasets generated during and/or analyzed during this study are available from the corresponding author on reasonable request.

## Code availability

Code for analyzes is available at https://github.com/naranoth/HCC-CTC-SCNA. The datasets used and/or analyzed during the current study are available from the corresponding author on reasonable request. Statistical analysis and visualization were performed in R (version 3.3.2).
