# Supplementary material for: Somatic copy number profiling from hepatocellular carcinoma circulating tumor cells
Source: NPJ Precis Oncol. 2020 Jul 2;4:16. doi: 10.1038/s41698-020-0123-0 (PMC7331695; doi:10.1038/s41698-020-0123-0)
Supplement: Supplementary file 1 — Supplemental Methods, Tables, and Figures [file 41698_2020_123_MOESM1_ESM.pdf]

Supplemental Figures and Tables

Supplemental Table 1. Clinical, Laboratory, Radiologic, Treatment and Pathologic Characteristics of the Patients (n = 10).

Supplemental Table 2. List of the 59 loci frequently amplified or lost in HCC as well as the associated study that reported the loci.

Supplemental Table 3. The 21 cancers grouped into 11 different cancer classes used in the final tumor origin classification model. The final model included 7002 individual samples from the 21 different cancer types.

Supplemental Table 4. List of the 31 cancer types included to create the SCNA-based classification model as well as the number of samples available for each cancer type.

Supplemental Figure 1. (A) STR analysis for patient H169 demonstrating that the CTC sample genotype does not match that of the primary tumor or blood. **(B)** Comparison of global copy number profiles for blood, peritumoral normal liver, CTCs, and primary tumor for patient H169.

Supplemental Figure 2. Array CGH of tumor and CTC samples for a subset of patients confirming the results of the NGS SCNA profiling.

Supplemental Figure 3. Spearman correlation matrix of CTC and primary tumor samples of the low-resolution whole genome sequencing copy number profiles for 250 kbp bins.

Supplemental Figure 4. (A) Principle component analysis of the whole blood, normal liver, primary tumor, and CTC (n = 7) samples for patient H195 revealed that most of the CTCs clustered closely with the primary tumor, while one CTC sample (CTC 7) clustered with the peritumoral normal and blood samples. **(B)** SCNA profiles for the primary tumor and all 7 CTC samples for patient H195. On examination of CTC 7's copy number profile, it clearly is closer to that of the germline DNA, and likely represents either a false positive CTC call or contamination. One additional CTC sample (CTC 4) clustered on its own, and examination of its copy number profile revealed significant discordance with all other samples as well. This finding is likely representative of problems during WGA or whole genome sequencing.

Supplemental Figure 5. t-SNE 2 dimensional plots for all samples from all cancer plotted for each cancer type alone on the entire t-SNE space. Some cancer types such as ESCA or BLCA demonstrate no clustering of patient samples while others such as TGCA or THCA demonstrate significant clustering.

Supplemental Figure 6. Cancer class prediction based on SCNA profile for each of the 29 lung CTC samples using the classification model. 23/29 (79%) of CTCs were correctly classified as being lung cancer while 6/29 (21%) were classified as being from

a liver source. 7/7 (100%) patients had at least one CTC classified as being from a lung tumor.

Supplemental Figure 7. Representative CTC images. **(A)** CTCs imaged on the silicon wire chip used for identification. **(B)** CTCs imaged on the PLGA chip used for laser microdissection. **(C)** Cells isolated by LMD are imaged on the cap to ensure complete isolation.

Supplemental Table 1. Clinical, Laboratory, Radiologic, Treatment and Pathologic Characteristics of the Patients (n = 10)

Characteristic	Data
Clinical	
Age, median (IQR)	65 (60 – 73)
Female, n (%)	3 (30)
Cirrhosis, n (%)	9 (90)
Diagnosis, n (%)*	
HCV	7 (70)
HBV	3 (30)
Cryptogenic	1 (10)
Laboratory	
Physiologic MELD, median (IQR)	7 (6 – 7)
AFP, most recent, median (IQR)	4.3 (3.6 – 8.7)
AFP, maximum, median (IQR)	4.9 (3.9 – 9.3)
HCC CTC count, median (IQR)	4 (2 – 11)
Radiologic tumor characteristics	
Tumor Location, n (%)	
Right Lobe	5 (50)
Left Lobe	2 (20)
Central	2 (20)
Retroperitoneum**	1 (10)
Maximum tumor diameter, median (IQR)	5.3 (3.8 – 7.5)
Pathologic Characteristics	
Tumor Diameter, median (IQR) ***	4.8 (3.6 – 7.9)
Microvascular invasion, n (%)***	5 (56)
Macrovascular invasion, n (%)***	1 (11)
PNI, n (%)***	0 (0)
Grade, n (%)	
Well	0 (0)
Moderate	6 (60)
Poor	4 (40)

* n = 1 patient with both HCV and HBV; ** n = 1, post-transplant recurrence undergoing retroperitoneal mass excision; *** n = 9 patients undergoing primary liver resection

Supplemental Table 2. List of the 59 loci frequently amplified or lost in HCC as well as the associated study that reported the loci.

Cytoband	Study	Cytoband	Study
11q13.3	Schulze, 2016	6p22.3	Kwon, 2013
12q22	Kan, 2013	6p24.2	Kwon, 2013
13q14.2	Schulze, 2016	6p25.3	Guichard, 2012
13q22.2	Kan, 2013	7p12.2	Guichard, 2012
13q33.1	Kan, 2013	7p15.2	Guichard, 2012
16p12.2	Kan, 2013	7p21.1	Guichard, 2012
16q13	Kan, 2013	7q31.2	Guichard, 2012
16q21	Kan, 2013	7q36.1	Woo, 2009
16q24.2	Kan, 2013	8p11.21	Roessler, 2012
17p11.1	Kan, 2013	8p12	Roessler, 2012
17p13.1	Kan, 2013	8p21.2	Roessler, 2012
17q21.2	Kan, 2013	8p22	Roessler, 2012
17q24.2	Kan, 2013	8q12.3	Kan, 2013
17q25.2	Zhang, 2015	8q13.1	Kan, 2013
1p12	Guichard, 2012	8q21.11	Kan, 2013
1p22.1	Guichard, 2012	8q21.3	Kan, 2013
1p33	Guichard, 2012	8q22.1	Kan, 2013
1p36.21	Guichard, 2012	8q23.1	Kan, 2013
1q21.3	Woo, 2009	8q24.11	Woo, 2009
1q22	Woo, 2009	8q24.21	Woo, 2009
1q42.11	Woo, 2009	8q24.22	Woo, 2009
20q12	Kan, 2013	8q24.3	Kan, 2013
20q13.12	Li, 2016	9p21.3	Guichard, 2012
20q13.2	Dauch, 2016	9q32	Guichard, 2012
21p11.2	Kan, 2013		
22q13.1	Kan, 2013		
3q26.31	Guichard, 2012		
5p15.33	Kan, 2013		
5p14.1	Kan, 2013		
6p12.2	Kan, 2013		
6p21.1	Chiang, 2008		
6p21.2	Chiang, 2008		
6p21.31	Chiang, 2008		
6p21.32	Guichard, 2012		
6p21.33	Chiang, 2008		

Supplemental Table 3. The 21 cancers grouped into 11 different cancer classes used in the final tumor origin classification model. The final model included 7002 individual samples from the 21 different cancer types.

**ENDO**<br>(ACC)	**LIVER**<br>(HCC, CHOL)	**GI**<br>(CRC, PDAC, STAD)
**OV**<br>(OV)	**PCPG**<br>(PCPG)	**KIDNEY**<br>(KICH, KIRC, KIRP)
**MALE**<br>(TGCT)	**LUNG**<br>(LUAD, LUSC)	**NERVOUS**<br>(GBM, LGG)
**MELANOMA**<br>(UVM)	**CNLOW**<br>(THYM, LAML, THCA, PRAD)	

Supplemental Table 4. List of the 31 cancer types included to create the SCNA-based classification model as well as the number of samples available for each cancer type.

Abbreviation	Cancer Type	Samples
BRCA	Breast invasive carcinoma	1080
COADREAD	Colon and rectal adenocarcinoma	616
OV	ovarian serous cystadenocarcinoma	579
GBM	glioblastoma multiforme	577
UCEC	uterine corpus endometrial carcinoma	539
KIRC	kidney renal clear cell carcinoma	528
HNSC	head and neck squamous cell carcinoma	522
LUAD	lung adenocarcinoma	516
LGG	low grade glioma	513
LUSC	lung squamous cell carcinoma	501
THCA	thyroid carcinoma	499
PRAD	prostate adenocarcinoma	492
STAD	stomach adenocarcinoma	441
BLCA	bladder urothelial carcinoma	408
LIHC	liver hepatocellular carcinoma	370
CESC	cervical squamous cell carcinoma and endocervical adenocarcinoma	295
KIRP	kidney renal papillary cell carcinoma	288
SARC	sarcoma	257
LAML	acute myeloid leukemia	191
ESCA	esophageal carcinoma	184
PAAD	pancreatic adenocarcinoma	184
PCPG	pheochromocytoma and paraganglioma	162
TGCT	testicular germ cell tumors	150
THYM	thymoma	123
ACC	adrenocortical carcinoma	90
MESO	mesothelioma	87
UVM	uveal melanoma	80
KICH	kidney chromophobe	66
UCS	uterine carcinosarcoma	56
DLBC	diffuse large b cell lymphoma	48
CHOL	choleangiocarcinoma	36

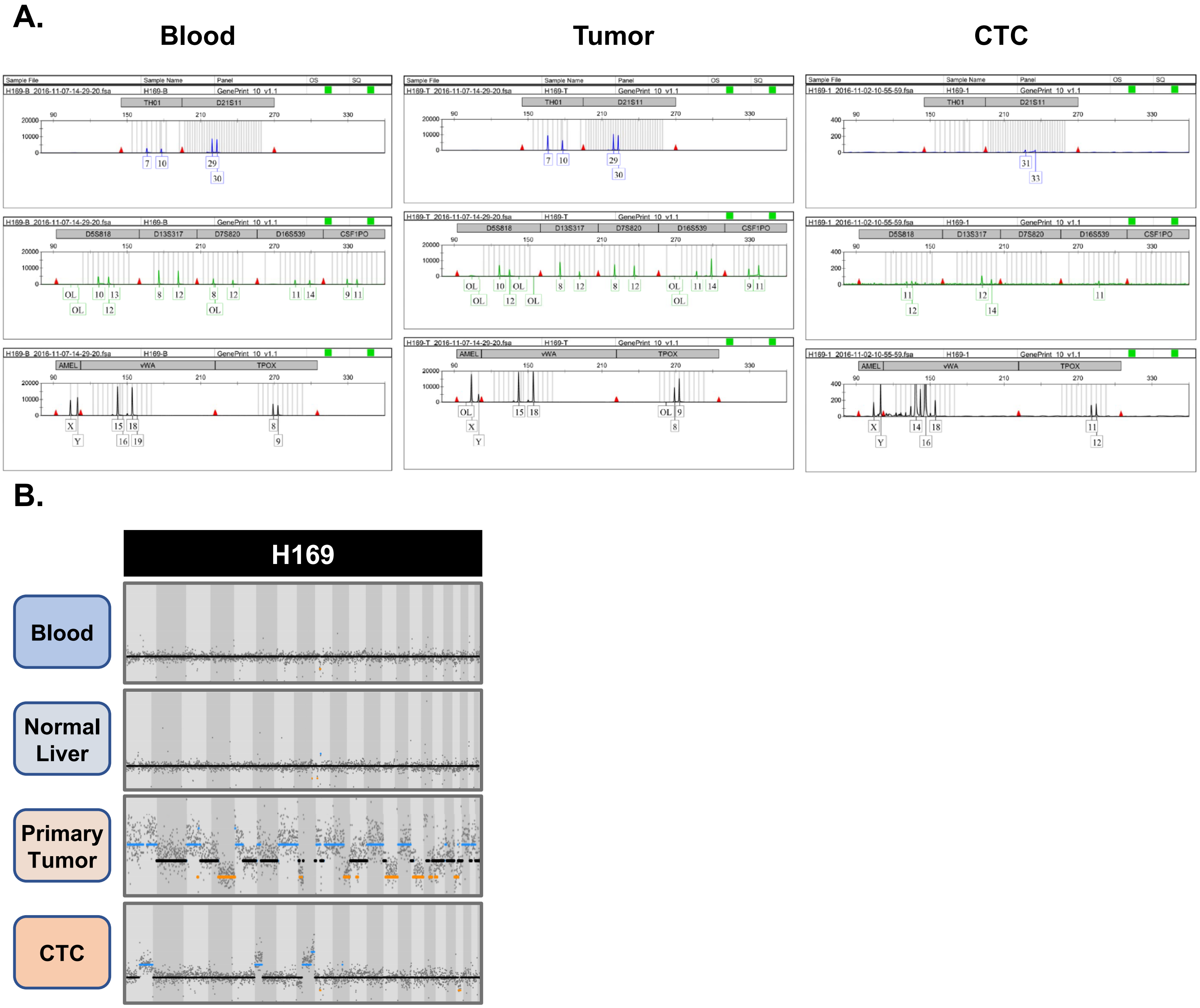

Supplemental Figure 1. (A) STR analysis for patient H169 demonstrating that the CTC sample genotype does not match that of the primary tumor or blood. **(B)** Comparison of global copy number profiles for blood, peritumoral normal liver, CTCs, and primary tumor for patient H169.

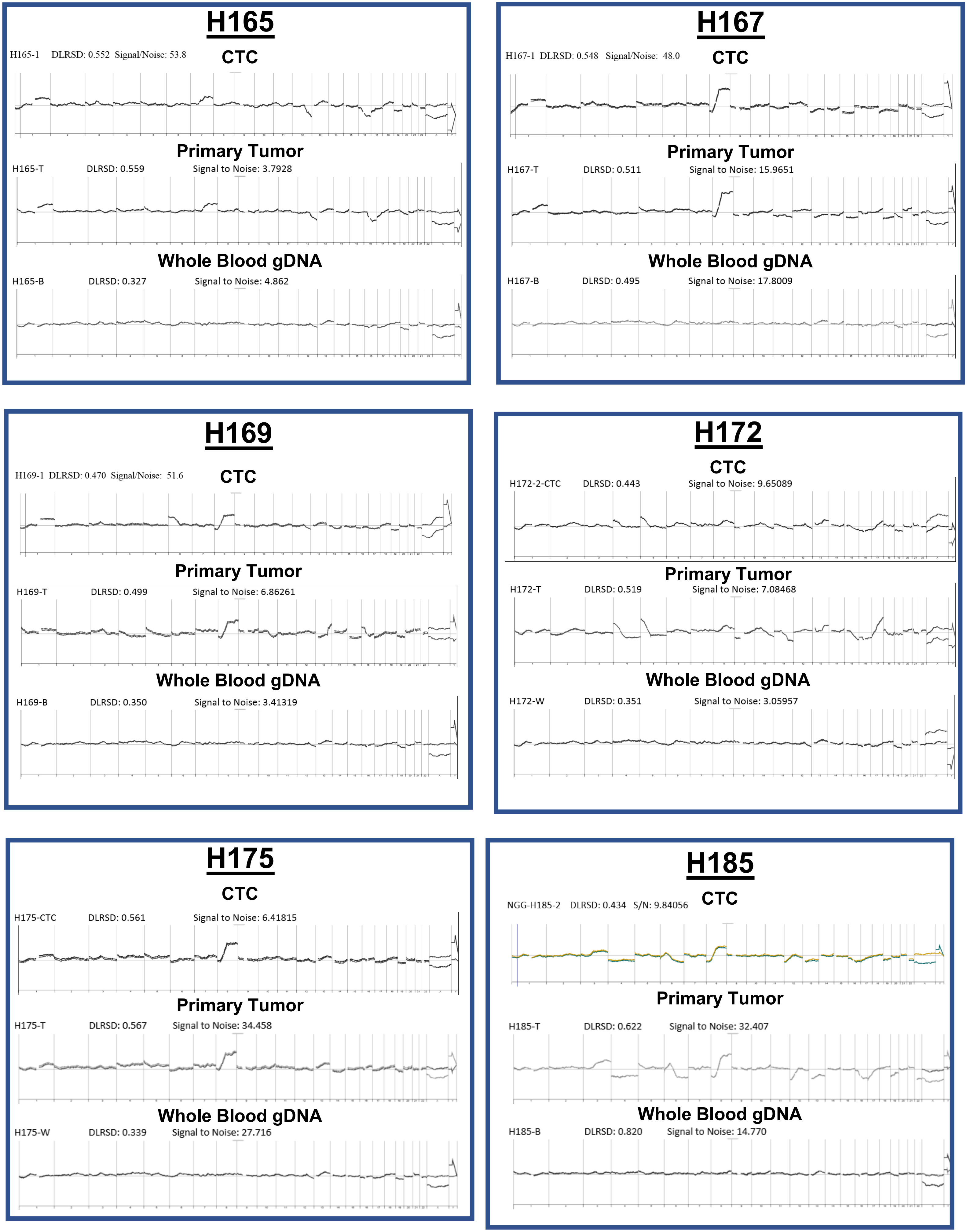

Supplemental Figure 2. Array CGH of tumor and CTC samples for a subset of patients confirming the results of the NGS CNV profiling.

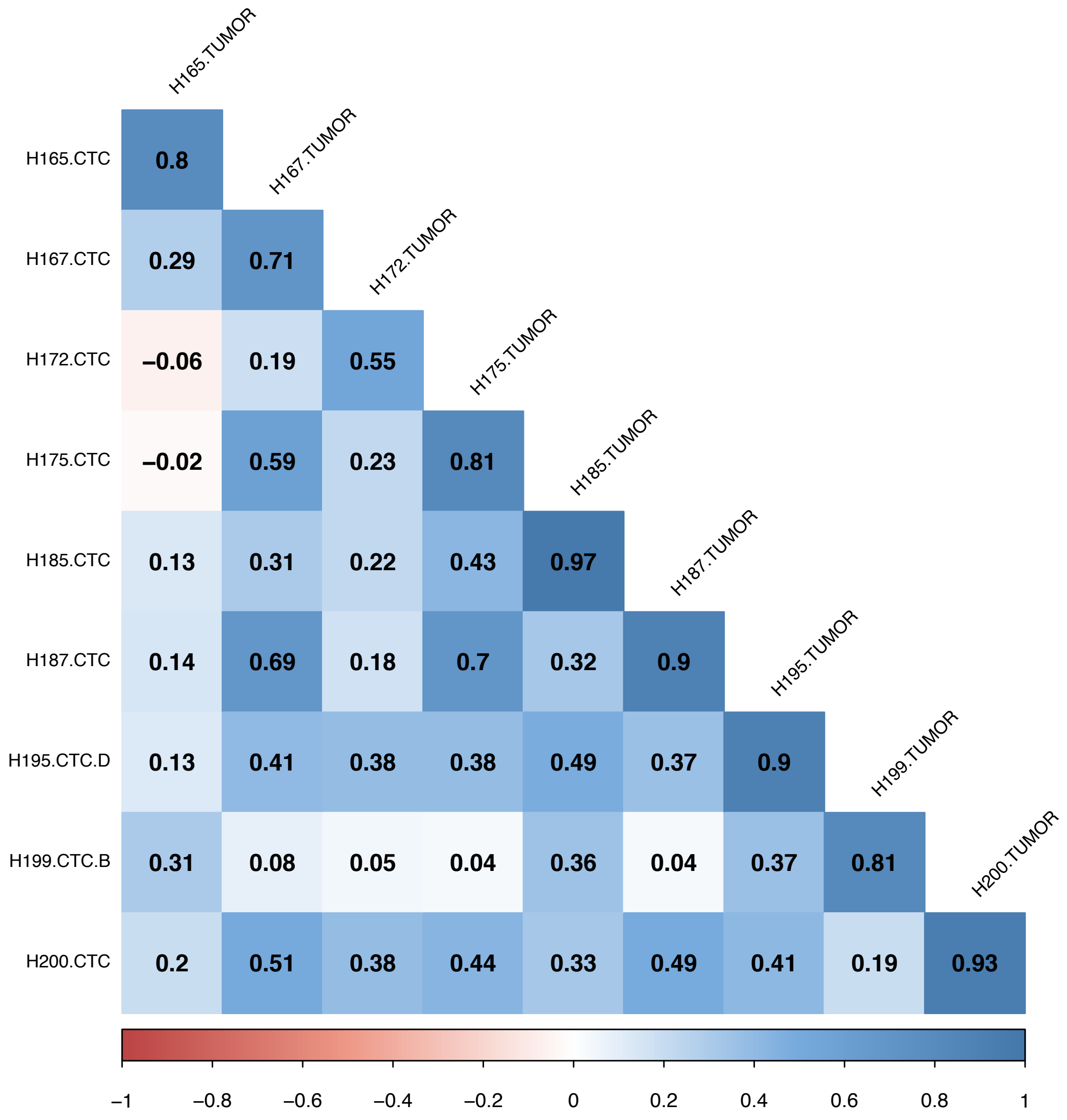

Supplemental Figure 3. Spearman correlation matrix of CTC and primary tumor samples of the low resolution whole genome sequencing copy number profiles for 250 kbp bins.

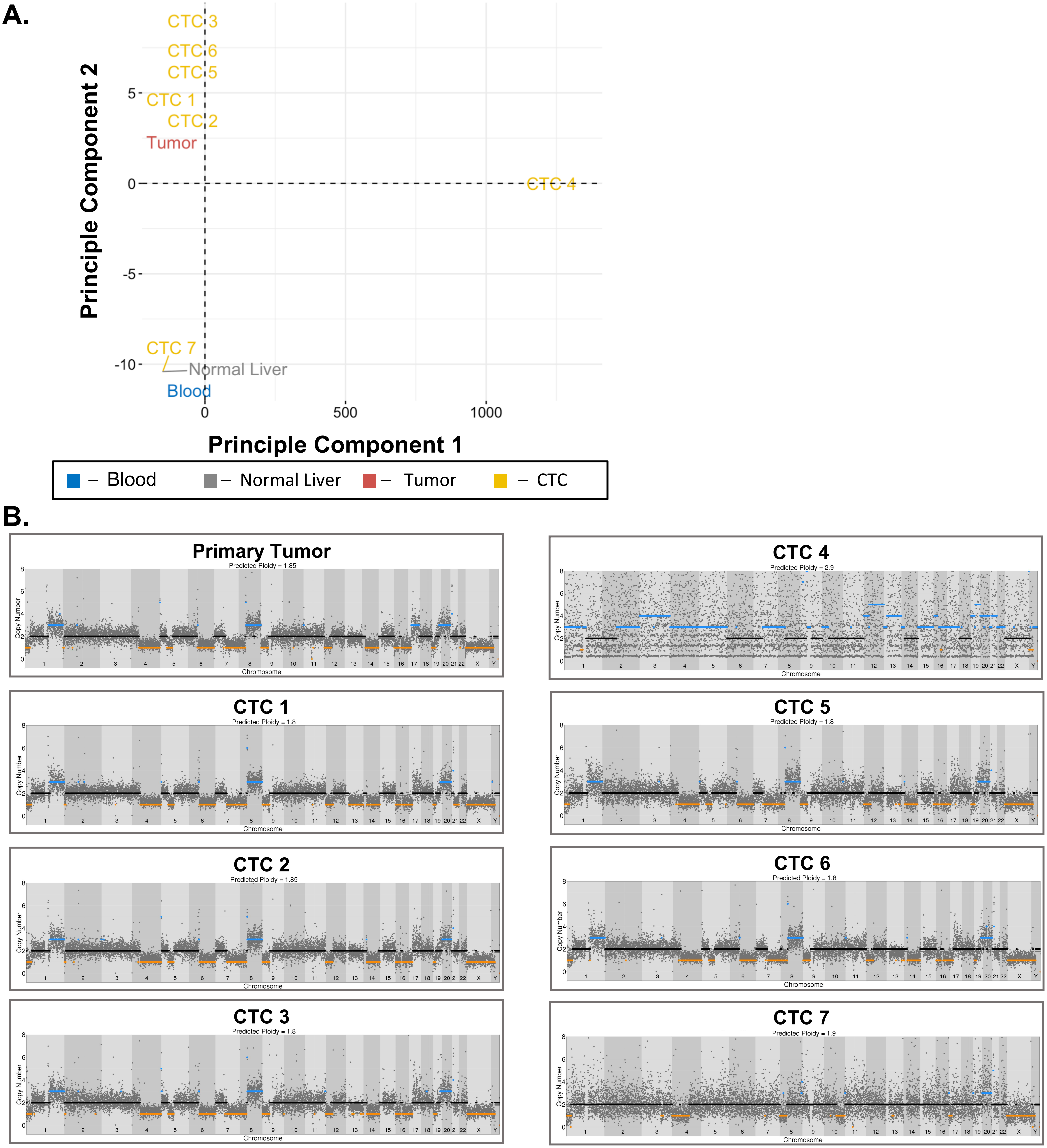

Supplemental Figure 4. (A) Principle component analysis of the whole blood, normal liver, primary tumor, and CTC (n = 7) samples for patient H195 revealed that most of the CTCs clustered closely with the primary tumor, while one CTC sample (CTC 7) clustered with the peritumoral normal and blood samples. **(B)** SCNA profiles for the primary tumor and all 7 CTC samples for patient H195. On examination of CTC 7's copy number profile, it clearly is closer to that of the germline DNA, and likely represents either a false positive CTC call or contamination. One additional CTC sample (CTC 4) clustered on its own, and examination of its copy number profile revealed significant discordance with all other samples as well. This finding is likely representative of problems during WGA or whole genome sequencing.

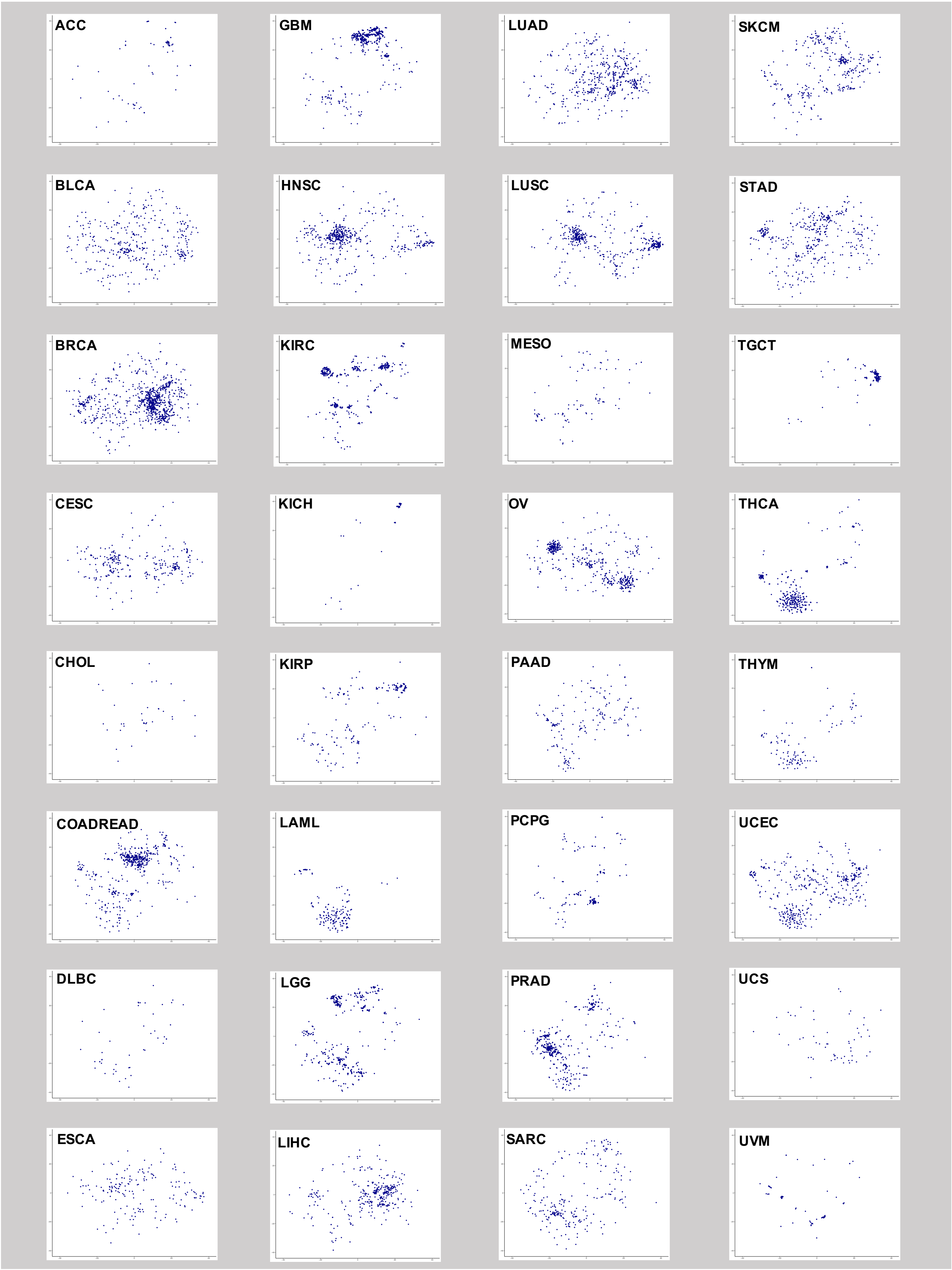

Supplemental Figure 5. t-SNE 2 dimensional plots for all samples from all cancer plotted for each cancer type alone on the entire t-SNE space. Some cancer types such as ESCA or BLCA demonstrate no clustering of patient samples while others such as TGCA or THCA demonstrate significant clustering.

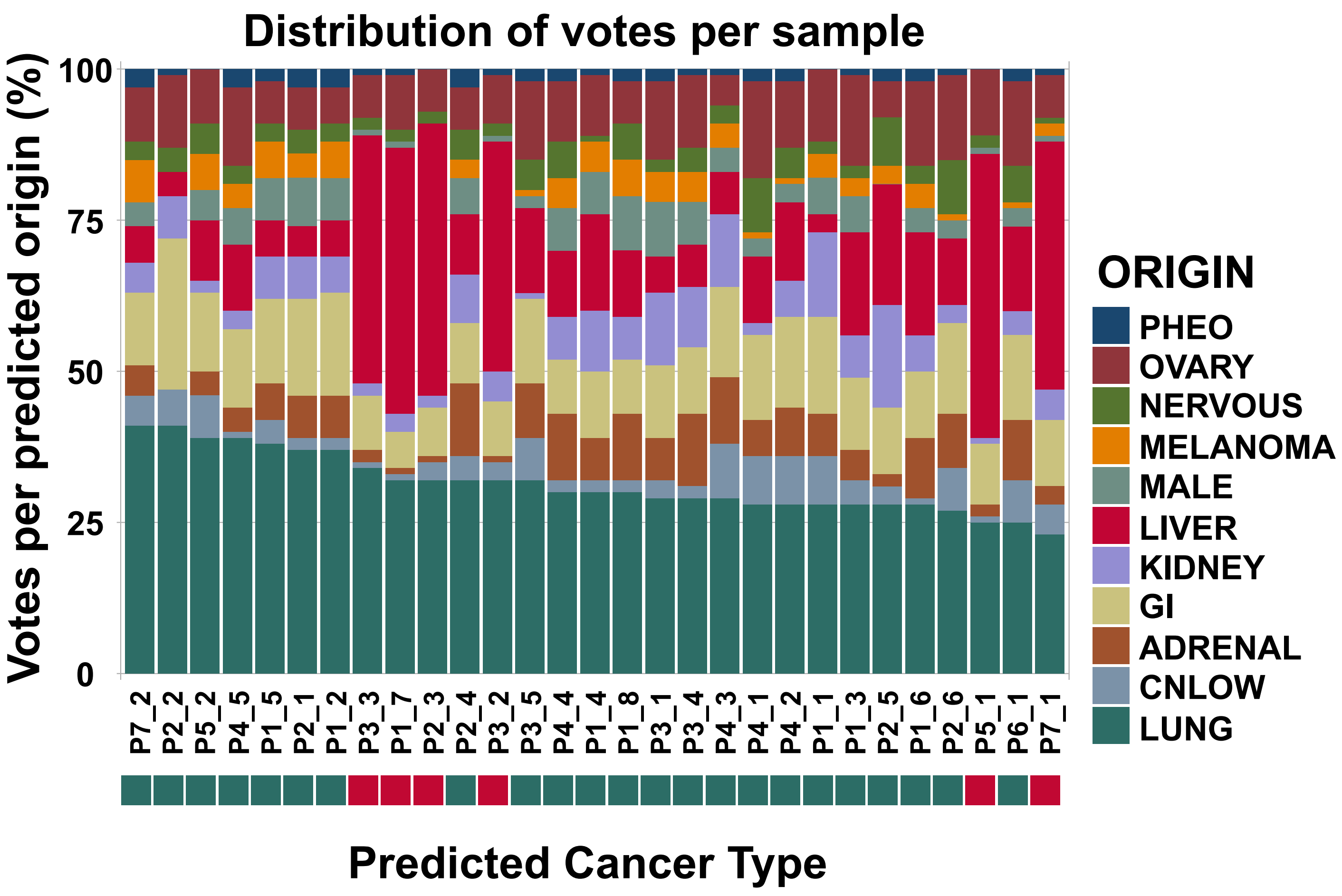

Supplemental Figure 6. Cancer class prediction based on SCNA profile for each of the 29 lung CTC samples using the classification model. 23/29 (79%) of CTCs were correctly classified as being lung cancer while 6/29 (21%) were classified as being from a liver source. 7/7 (100%) patients had at least one CTC classified as being from a lung tumor.

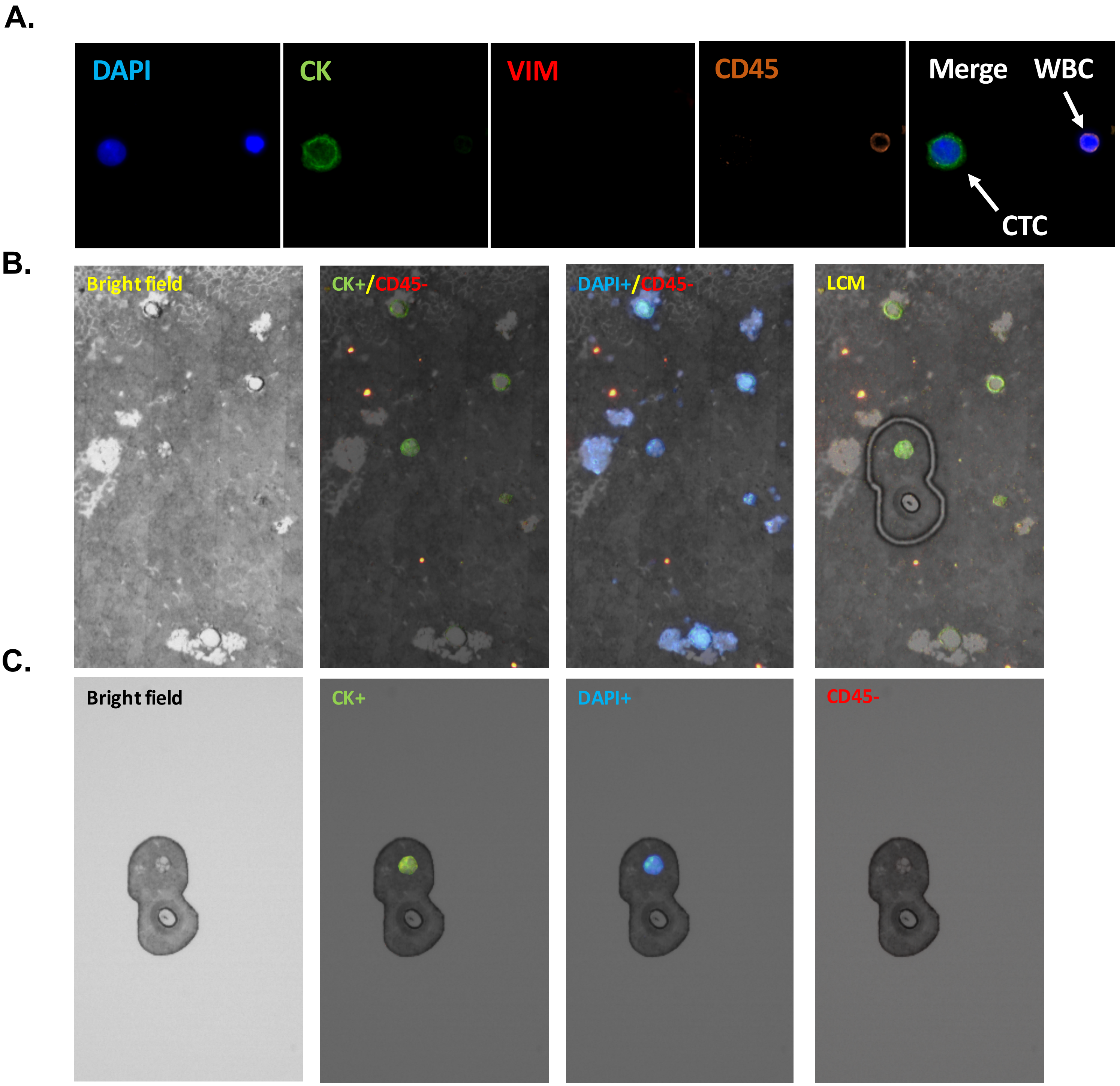

Supplemental Figure 7. Representative CTC images. **(A)** CTCs imaged on the silicon wire chip used for identification. **(B)** CTCs imaged on the PLGA chip used for laser microdissection. **(C)** Cells isolated by LMD are imaged on the cap to ensure complete isolation.
